# Bony fish genomes: Status and gaps

**DOI:** 10.1111/jfb.70402

**Published:** 2026-03-18

**Authors:** Noelia Pérez‐Pereira, Carmen Bouza, Paulino Martínez, Diego Robledo

**Affiliations:** ^1^ Departamento de Zoología, Genética y Antropología Física Universidade de Santiago de Compostela Santiago de Compostela Spain; ^2^ The Roslin Institute and Royal (Dick) School of Veterinary Studies, University of Edinburgh Easter Bush Midlothian UK

**Keywords:** assembly quality, bony fish, comparative genomics, genomes, phylogeny

## Abstract

Bony fish constitute an exceptionally species‐rich group of aquatic vertebrates, comprising more than 95% of all living fish. The adaptive processes on the diversity of environments they inhabit make them a highly diverse group from taxonomic, morphological and evolutionary standpoints. Furthermore, among their members we find species of high economic value for global aquaculture and fisheries. This has attracted the attention of multiple research fields, from ecology or evolutionary biology to comparative genomics. However, deep biological or evolutionary studies require suitable genomic resources, particularly high‐quality reference genomes, across multiple species and taxonomic groups. Here, we revise the current status of open access bony fish genome assemblies, with special emphasis on existing gaps throughout their phylogeny. Recently, there has been a boom of information and up to 1744 genomes are available, largely released in the last 5 years. They are distributed across 67 of the 72 recognized orders, where Cichliformes, Perciformes, Siluriformes, Cypriniformes and Cyprinodontiformes appear overrepresented, and across 268 families out of the 514 recognized. Overall, these figures cover around 5% of all described bony fish species. Despite five orders and 14 order‐level *incertae sedis* families (mostly percomorphs) lacking genomic representation, high‐throughput sequencing data for most gaps are available in the Sequence Read Archive repository of the National Center for Biotechnology Information. Future sequencing efforts aimed at improving assembly quality and filling the gaps in the fish phylogeny, in parallel to the evolution of sequencing and assembly technologies, will enhance our ability to answer open evolutionary questions in bony fish and provide genomic tools to boost fish production.

## 1 FISH: THE MOST DIVERSE VERTEBRATES

Fish constitute a broad non‐taxonomically formal category that covers more than half of the known living vertebrates. They can be defined as aquatic chordates displaying gills and fin‐shaped limbs (Facey et al., 2022), and among the three major groups, cartilaginous fish (sharks, rays and chimaeras), jawless fish (lampreys and hagfish) and bony fish, the latter is the most species‐rich. Bony fish comprise more than 95% of all known fish (Diana & Höök, 2023), including coelacanths, lungfish and ray‐finned fish. Both coelacanths and lungfish are considered bony fish showing primitive features within the superclass Sarcopterygii (i.e. they are closer to tetrapods than to other fish; Schartl et al., 2024), each with only one extant order (Coelacanthiformes and Ceratodontiformes, respectively). In contrast, ray‐finned fish (superclass Actinopterygii) have undergone intense diversification throughout their history, notably the Triassic radiation and the rapid radiation of percomorphs in the Late Cretaceous (Hughes et al., 2018; Romano et al., 2016). Actinopterygii comprehend more than 32,000 species, mostly organized into the 70 currently recognized orders (Betancur‐R et al., 2017), which makes them the largest group of living fish.

The high interest in bony fish goes beyond their economic, nutritional and social value to humans. For example, farmed finfish production increased by 170% since 2010, registering a global production of 53.3 million metric tons in 2019 (Verdegem et al., 2023). This growth is being driven by strategic priorities that support the sustainable management of farmed and wild aquatic genetic resources (FAO, 2022). Besides that, bony fish inhabit a wide variety of marine and freshwater environments, including variation in temperature, pressure, oxygen concentration, light and even dryness. Consequently, they constitute a highly diverse group from taxonomic, morphologic, physiologic, behavioural and life‐history perspectives (Facey et al., 2022). This has attracted the interest of multiple fields, including ecology and evolutionary biology.

For instance, the three‐spine stickleback species complex is classically considered an evolutionary model system for the study of sexual selection, as well as parallel adaptation, due to its natural history characterized by repeated colonization events from marine to freshwater environments (McKinnon & Rundle, 2002). Fish have also been extensively investigated to study the evolution of sex determination, which is highly diverse in this group and includes genetic and environmental determination (mainly temperature), or interactions between them (Martínez et al., 2014). To date, up to 21 sex‐determining genes have been identified in 114 teleost species, but the screening of new chromosome‐level genomes is providing a much more detailed picture, with new unexpected genes and pathways identified (Kitano et al., 2024). On the other hand, organisms such as zebrafish, medaka or trout are being widely used as models for toxicology (MacRae & Peterson, 2023; Shahjahan et al., 2022) and biomedicine (e.g. cancer or infectious diseases; Kobar et al., 2021; Ernst et al., 2023), as well as for development research (Lawson & Wolfe, 2011) or aging (Gerhard, 2007), among other fundamental and applied relevant topics.

## 2 FISH GENOMICS

In‐depth fish evolutionary studies are greatly facilitated by the availability of consistent genome assemblies across multiple species and groups. Much of the scientific progress achieved over the last few years in bony fish has been possible thanks to the development of genomic sequencing technologies, especially long‐read sequencing, new scaffolding methods and appropriate bioinformatic approaches. Together, these advances have opened the era of comparative genomics (e.g. see reviews by Froschauer et al., 2006; Spaink et al., 2014; Ahmad et al., 2022). The initial whole‐genome sequencing of fugu (*Takifugu rubripes*), pufferfish (*Tetraodon nigroviridis*), zebrafish (*Danio rerio*), medaka (*Oryzias latipes*) and the three‐spined stickleback (*Gasterosteus aculeatus*), followed by a growing list of fish species (384 genomes counted in the National Center for Biotechnology Information [NCBI] database in 2020; Ahmad et al., 2022), enabled the study of the genome organization and its evolution (e.g. Kushwaha et al., 2023; Li et al., 2022). This information opened the door to analysing the evolutionary relationships among vertebrates through orthology, thus contributing to a better understanding of the mechanisms of adaptation and evolution (Hughes et al., 2018). It has also enabled the identification of conserved coding and regulatory regions, and the distribution of genome repeats and structural variants (Reinar et al., 2023; Venkatesh & Yap, 2005).

In addition, the identification of genome duplications, involving either the whole genome or specific genomic regions or gene families, laid on the table their functional implications and roles on adaptation. This is exemplified by studies of gene families such as the *hox* gene clusters or the major histocompatibility complex ‘MHC’ (Ahmad et al., 2022; Glasauer & Neuhauss, 2014). Currently, up to three main whole genome duplications (WGD) have been identified involving bony fish, two of them occurring at the early stages of vertebrate's evolution (1R and 2R, ~490–530 million years ago, 2R relevant for body plan and species diversification; Yu et al., 2024), one teleost‐specific duplication (Ts3R, ~300 million years ago; Inoue et al., 2015; Conant, 2020) and one sturgeon‐specific duplication (family Acipenseridae; Ars3R, 180 million years ago; Du et al., 2020; Höhne et al., 2021). A fourth, more recent, lineage‐specific WGD event involves salmonids (Ss4R, around 80 million years ago; Lien et al., 2016), suckers (family Catostomidae; Cat4R, around 25.2 million years ago; Krabbenhoft et al., 2021) and cyprinids (family Cyprinidae; Cs4R, around 14 million years ago; Chen et al., 2019; Xu et al., 2019; Ren et al., 2022).

With the decreasing sequencing costs, the possibility of sequencing up to 10,000 vertebrate genomes (approximately one genome per genus) was opened in 2009 by projects such as the Genome 10K, of which almost half were expected to belong to fish species (see review by Bernardi et al., 2012). The following year, a pilot project was launched with the aim of sequencing 101 vertebrates, including 28 fish, and discussions were held on the criteria for selecting future targets. The focus was not only on the species' commercial relevance in fisheries and aquaculture or on its threatened status, but also on the importance of filling gaps in the phylogeny.

Today, new fish genomes are constantly being added to open‐access databases such as the NCBI (Sayers et al., 2024) and Ensembl (Harrison et al., 2024), fuelled by new initiatives and projects (e.g. the 10,000 Fish Genomes Project ‘Fish10K’, The Vertebrate Genomes Project ‘VGP’, or the Fish‐T1K project for transcriptomic data; Sun et al., 2016; Fan et al., 2020; Rhie et al., 2021). This wealth of available genomic data makes it possible to address outstanding evolutionary questions in bony fish. For instance, uncertainties still persist in the phylogenetic tree of bony fish despite previous attempts to solve them (Dornburg & Near, 2021; Hughes et al., 2018). The present study aims to provide a snapshot of the genomic resources in bony fish currently available in the NCBI and Ensembl databases, showing the existing gaps in genomic information throughout the phylogenetic tree. This information will be useful to evolutionary biologists planning studies along the teleost phylogeny and will hopefully focus new sequencing efforts.

## 3 AN OUTBURST OF NEARLY COMPLETE FISH GENOMES

**FIGURE 1:**
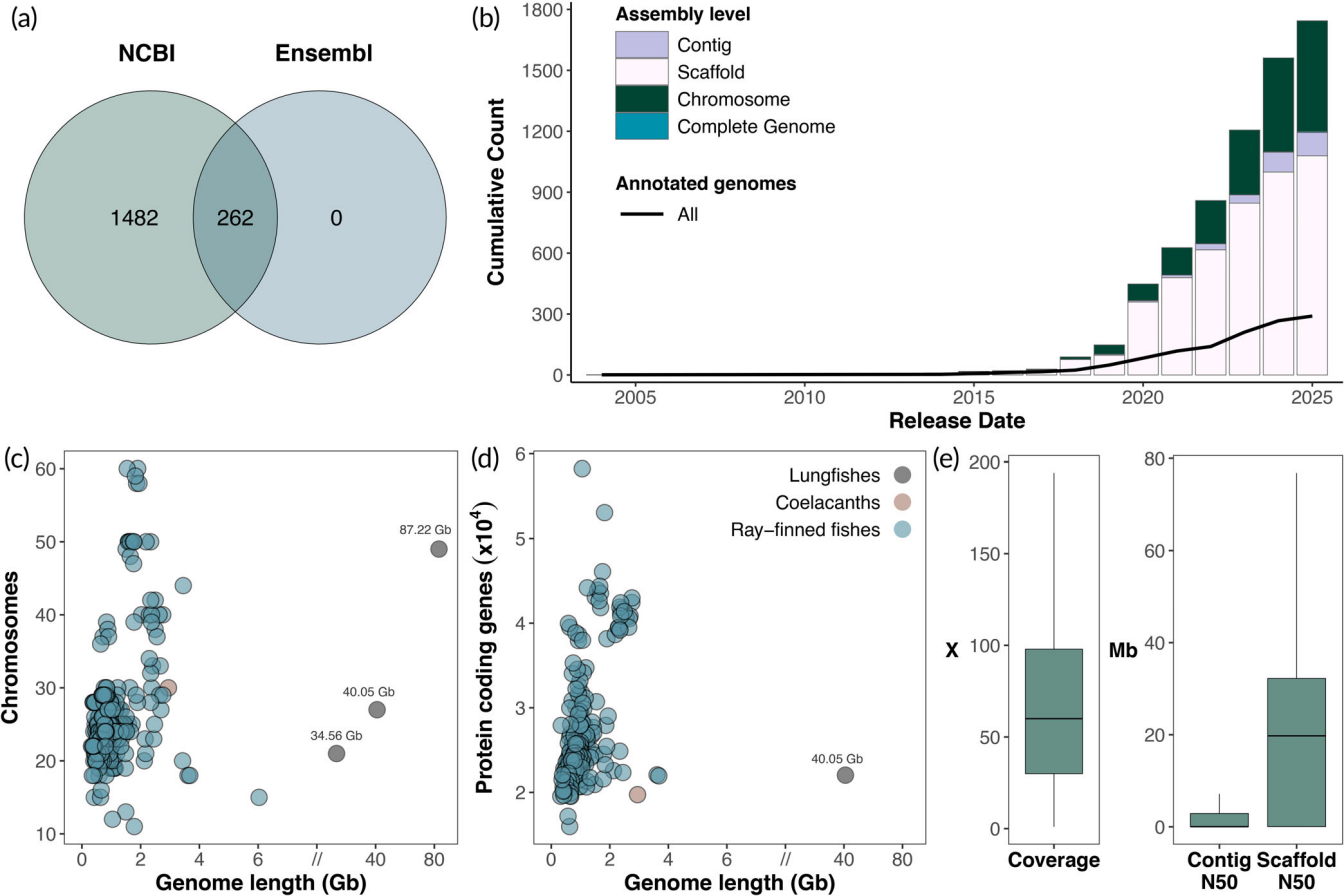
Genome assemblies in bony fish. (a) Number of shared and specific species in the NCBI and Ensembl (including Ensembl Rapid Release) databases. (b) Cumulative number of genomes released over time, categorized according to their assembly level. The number of annotated genomes is indicated by the solid line. (c) Relationship between (haploid) chromosome number and genome length (Gb). (d) Relationship between the number of protein coding genes and genome length (Gb). (e) Three genome assembly statistics, genome coverage (x) (data for 1742 species), contig N50 (1744 species) and scaffold N50 (1744 species). The boxes represent the 50% of the estimates, i.e. the interquartile range, and the median is indicated by the horizontal solid line. Outliers were removed for legibility. Data for (b)–(e) were obtained from the NCBI database.

Genome assemblies for a total of 1744 bony fish species were found in the NCBI database, 262 of them also found in Ensembl or Ensembl Rapid Release database (Figure 1a and File S1). The majority are classified within the superclass Actinopterygii (99.77%), while the remaining 0.23% are coelacanths and lungfish, in the superclass Sarcopterygii. This is not surprising since both coelacanths and lungfish constitute small groups, each with a single extant order and two and six species described in the Catalogue of Life, respectively. It is noteworthy that the 80 genomes ascribed to unclassified species within a genus pertained mostly to cichlids. Also, 21 genomes were allocated to subspecies, three classified as below‐species or with no rank and four as hybrids. In any case, the number of available fish genomes is markedly higher than in previous reports, 384 genomes were recorded in 2020 by Ahmad et al. (2022) (312 teleosts and 72 non‐bony fish) and 583 finfish genomes were reported by Randhawa and Pawar (2021) by the end of the same year. Hotaling et al. (2021) also counted 685 fish genomes at the beginning of 2021. In addition, among the available animal genomes, they found a clear bias in favour of vertebrate taxa, including actinopterygians which, along with tetrapods, appear overrepresented, denoting the exceptional efforts dedicated to fish genomic resources.

Recently, the boom of fish genomic sequencing has made it possible to categorize reference genomes in many fish species. These include chromosome level assemblies, clearly increasing over time, as well as complete genome assemblies with no gaps or unplaced/unlocalized scaffolds, which are starting to be released (currently five fish complete genomes: *Acanthopagrus schlegelii*, *Cephalopholis sonnerati*, *Danio rerio*, *Ilisha elongata* and *Triplophysa yaopeizhii*) (Figure 1b). Genome size of bony fish varies widely among taxa, the largest genomes being found in lungfish (53.94 gigabases [Gb] of total sequence length on average) and the smallest in finfish of the order Tetraodontiformes (0.42 Gb on average) (Figure S1). A size reduction trend can be observed along the phylogenetic tree, from larger genomes in basal clades to smaller genomes in more recently diverged groups, as noted before (Facey et al., 2022). Previous works have associated genome length with intron size and proportion of repetitive elements (Villarreal et al., 2024; Yuan et al., 2018), as well as transposable elements (TEs; Schartl et al., 2024). For instance, the South American lungfish (*Lepidosiren paradoxa*) has the largest sequenced animal genome to date. With around 91 Gb in length divided into 19 chromosomes and a total of 49 assembled pieces, this species exhibits the highest reported rate of diploid genome expansion. The still active massive expansion has been linked to the suppression mechanisms preventing TE accumulation (Schartl et al., 2024). A negative correlation has also been found between the effective population size and genome size, freshwater species usually having smaller effective size and larger genomes than marine species (Yi & Streelman, 2005). Genome size can also be affected by marine water depth (Medeiros et al., 2022). Recently, Kushwaha et al. (2023) detected a positive correlation between genome size and body length for 51 teleost fishes, but no correlation was found with chromosome number, which was more closely associated with ploidy level and chromosomal rearrangements.

Among the 1744 available fish genomes, we found chromosome number information for 550 of them, ranging from *n* = 11 (*Umbra pygmaea*, Esociformes) to *n* = 60 chromosomes (*Acipenser ruthenus* and *Polyodon spathula*, Acipenseriformes), with an average of 25.41 (Figure 1c). The highest chromosome number was found in the orders Acipenseriformes (average *n* = 59.00), Stomiiformes (only one species with *n* = 37), Salmoniformes (average *n* = 36.80) and Ceratodontiformes (average *n* = 32.33) (Figure S2). Previous studies pointed to 24 as the ancestral haploid chromosome number of ray‐finned fishes, and around 12 for its last common ancestor and for sarcopterygians (Facey et al., 2022; Glasauer & Neuhauss, 2014).

Regarding assembly quality, the genome coverage, contig N50 and scaffold N50 varies widely among species, with an average coverage of 114.86x (median 60.00x), an average contig N50 of 4.95 Mb (median 0.02 Mb), and an average scaffold N50 of 23.09 Mb (median 19.76 Mb) (Figure 1e). Overall, only 34.40% of genomes have a contig N50 bigger than 1 Mb (64.62% for scaffold N50), and among those reference genomes not assembled at chromosome level, only 10.97% (48.41% for scaffold N50).

Gene annotation has also experienced a boost recently, albeit at a slower rate (Figure 1b, solid line). Currently, only 290 bony fish genomes (16.49%) have gene annotation. The number of protein‐coding genes ranges from 15,965 (*Nibea albiflora*, Sciaenidae) to 58,247 (*Cirrhinus mrigala*, Cypriniformes), with an average of 26,338.54 (Figure 1d). As for genome size, a trend of decreasing gene number can be observed along the phylogenetic tree (Figure S3), in accordance with the correlation found previously between genome size and gene count in eukaryotes (Elliott & Gregory, 2015). Similarly, the number of non‐coding genes, available for 248 genomes, ranges from 24 (*Knipowitschia caucasica*, Gobiiformes) to 48,441 (*Carassius gibelio*, Cypriniformes). The number of annotated pseudogenes (245 genomes) also varies considerably among taxa, from one (*Cirrhinus mrigala*, Cypriniformes, *Gymnothorax javanicus*, Anguilliformes, and *Umbra pygmaea*, Esociformes) to 16,891 (*Leuresthes tenuis*, Atheriniformes).

**FIGURE 2:**
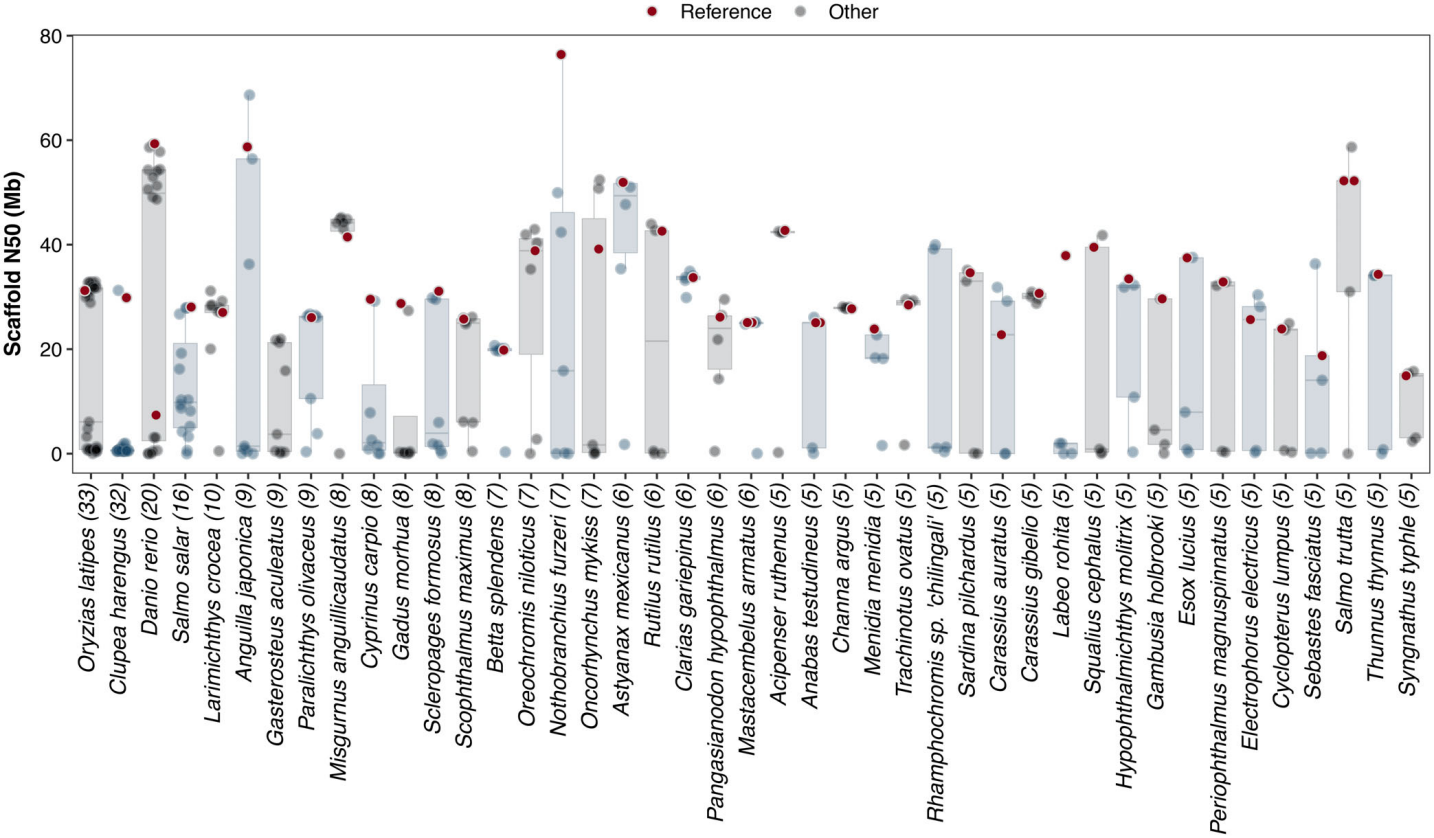
Assembly quality, measured as scaffold N50 (Mb), for each of the genomes deposited at NCBI and Ensembl per organism. Only organisms with five or more genomes are shown. The boxes represent the 50% of the estimates, i.e. the interquartile range, and the median is indicated by the horizontal solid line. Each circle represents a genome (reference genome in red). The total number of genomes per organism is also indicated in parentheses.

At the organism level, several species have been found to be particularly enriched with genomic data. Of the 1846 organisms included in the NCBI and Ensembl databases (including hybrids, subspecies and undefined species; File S2), 38.14% have more than one assembly available (File S3), and 2.33% have between five and 33 assemblies (Figure 2). The quality of the multiple genomes per organism varies widely, but those categorized as reference generally fall close to or above the third quartile (Figures 2 and S4). Seventy‐five per cent of these reference genomes have a scaffold N50 above 25.66 Mb (Figure 2) and a contig N50 above 2.53 Mb (Figure S4). Given the accelerating rate at which new genomes are being released, these genomes will soon be outdated and replaced by improved assemblies. Note that most of the species in Figure 2 are of high commercial value in fisheries, sport fishing and aquaculture. Examples include the medaka (*Oryzias latipes*), which has the largest number of published assemblies, as well as the Atlantic herring (*Clupea harengus*), Atlantic salmon (*Salmo salar*) and the large yellow croaker (*Larimichthys crocea*). Others are well‐known model organisms in studies of evolution and adaptation, including adaptation to terrestrial life and dark environments, as well as developmental and aging research.

## 4 TOWARDS FULL COVERAGE OF THE BONY FISH PHYLOGENY

**FIGURE 3:**
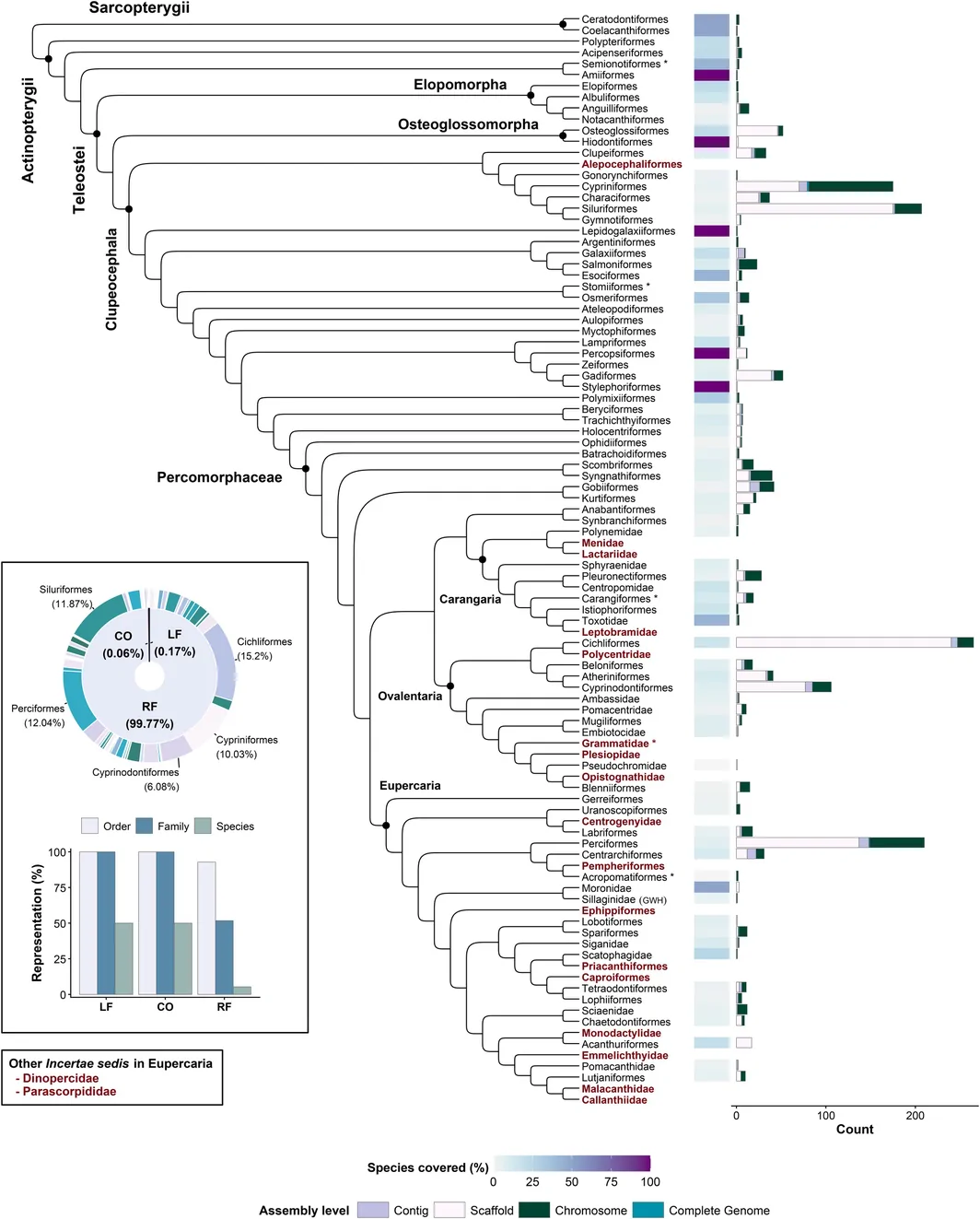
Distribution of genomic data among orders (or families in case of order‐level *incertae sedis*). The phylogenetic tree was reconstructed from Betancur‐R et al. (2017). The (approximate) percentage of species represented with genomic data is indicated by colour in the heatmap column. The number of species currently recognized per order or family was taken from the Catalogue of Life (see Material and Methods). The total number of released genomes per order or family, and categorized according to their assembly level, is indicated in the bar plot. Orders or families without available genomic data are also marked in red. Box: Global view of genomes available and representation. Upper plot: Percentage of ray‐finned fish (RF), coelacanths (CO) and lungfish (LF) with genomic data (inner pie plot), and the five most represented orders within ray‐finned fish (outer donut plot). Lower plot: Percentage of orders, families and species represented with genomic data. The number of orders and families currently recognized was taken from Betancur‐R et al. (2017), and the total number of species from the Catalogue of Life. Semionotiformes*: Lepisosteiformes in Betancur‐R et al. (2017). Stomiiformes*: Stomiatiformes in Betancur‐R et al. (2017). Carangiformes*: Does not constitute a monophyletic group (position in the present tree corresponds to the family Carangidae). Grammatidae*: Does not constitute a monophyletic group (position in the present tree corresponds to the genus *Lipogramma*). Acropomatiformes*: Not recognized in Betancur‐R et al. (2017) (families included within the order Pempheriformes, and Dinolestidae as order‐level *incertae sedis* in Eupercaria).

While the number of bony fish genome assemblies available has doubled in the last 3 years, they still represent only 5.36% of the species recognized in the Catalogue of Life. Nonetheless, these are roughly spread throughout most of the bony fish phylogeny, with extant genomic data for 67 of the 72 orders recognized by Betancur‐R et al. (2017) (93.05%). Among them, 65 orders correspond to ray‐finned fish, being the five most represented Cichliformes, Perciformes, Siluriformes, Cypriniformes and Cyprinodontiformes in decreasing order, covering 55.22% of all genomes (see box in Figure 3). At the family level, 268 of the 514 recognized families (Betancur‐R et al., 2017) are represented (52.14%) (box in Figure 3). As expected, the representation of orders, families and species of coelacanths and lungfish is greater than that found globally for actinopterygians. Lungfish, for example, are key organisms for understanding the genetic and molecular mechanisms behind the vertebrates' water‐land transition (Schartl et al., 2024), so the availability of their sequenced and assembled genomes becomes a very valuable tool.

Figure 3 shows a histogram distribution of genomic data along the phylogenetic tree of bony fish. The biggest bars displayed by the orders Cichliformes, Perciformes, Siluriformes, Cypriniformes and Cyprinodontiformes stand out, as noted above, despite the rather low percentage of genomes in the recognized species (3.96%–15.57%; Cichliformes being the highest). This pattern is expected, as these orders are among the 10 most species‐rich, comprising around 1700 species in cichlids, more than 2900 in Perciformes, over 3700 in Siluriformes, more than 4400 in Cypriniformes and around 1300 in Cyprinodontiformes.

Cichlids are a highly diverse group considered a model for multiple research fields, such as ecology or evolution, due to their history of explosive adaptive radiations, largely associated with the colonization of lake environments, and their exceptional phenotypic diversity (e.g. colour, body shape or craniofacial and dental morphology; Santos et al., 2023). The interplay of natural and sexual selection, ecological adaptation to different trophic environments (including parallel adaptation) and phenotypic plasticity have distinctively shaped this group (Santos et al., 2023). These features explain the huge investment in genome sequencing in this group. For instance, cichlids from the Great Lakes of the East African rift have captured the attention for being a biodiversity hotspot and in fact, Lake Victoria cichlids have one of the highest speciation rates among vertebrates (Santos et al., 2023). Lake Malawi cichlids also underwent a recent major species radiation, and the sequencing of all major lineages has improved our understanding of the genomic processes underlying rapid species diversification. Specifically, signatures of both adaptive introgression and independent selection were found in visual and oxygen transport genes in deep‐related species (Malinsky et al., 2018), and remarkable examples on sex determination changes associated with sexual conflict were provided (Roberts et al., 2009). A similar diversification has been reported in the American Neotropical cichlids in part associated with the profusion of small crater lakes in Central America (Arbour & López‐Fernández, 2014). Rockfish (genus *Sebastes*, Perciformes) is also a highly diverse group, with over 100 species across the North Pacific Ocean. Recent de novo genome sequencing and assembly of 88 species has shed light on the genetic basis of their extreme lifespan diversity, which includes some of the longest‐lived vertebrates (Kolora et al., 2021).

Several commercially important species can also be found within the previous species‐rich orders. For instance, the cichlid Nile tilapia is among the five most important fish aquaculture species worldwide (FAO, 2024). Other species, such as carp (Cypriniformes), catfish (Siluriformes), perch or groupers (Perciformes), are also among the fish species with the highest aquaculture production (Basurco et al., 2011; Gisbert et al., 2022; Verdegem et al., 2023). On the other hand, guppy, mollies or swordtails (Cyprinodontiformes) are well‐known aquarium species (Ghedotti, 2000). The order Gadiformes is also one of the most species‐rich and encompasses a considerable number of genomes. It includes economically relevant species, such as the Atlantic cod (*Gadus morhua*) and the European hake (*Merluccius merluccius*).

In contrast to cichlids, the large genome sequencing effort in other groups is the result of many independent projects focused not only on their commercial interest, but in different evolutionary features, such as the allotetraploidy of common carp (Xu et al., 2019). Cyprinodontiformes have also been used as models to study the convergent evolution of viviparity (Yusuf et al., 2023), as well as the ability to tolerate land environments, being one of the most diverse orders containing the so‐called amphibious fish (Wright & Turko, 2016). The order Gadiformes has also attracted attention in studies focusing on the evolution of the immune system in teleost due to the successive loss of immunological components characteristic of all species of the order (Malmstrøm et al., 2016; Solbakken et al., 2016). They have also been investigated in relation to adaptation to harsh environments, such as deep‐sea (Bo et al., 2022) and Arctic waters (Maes et al., 2025).

Genome research is essential for a better understanding of the evolution and the biology of the species, as well as to uncover the genomic basis of adaptation to environmental changes to promote sustainable fisheries (Andersson et al., 2024; Layton et al., 2024). It is also important for disentangling the genetic architecture of productive traits for breeding programs, such as reproduction, fecundity, performance, growth rate or disease resistance (Jin et al., 2016). Directly or indirectly, genomic efforts also echo the growing research interest in other fields, for instance toxicity and tolerance studies in catfish (Kar & Senthilkumaran, 2024), or taxonomy applied to biodiversity and conservation (Thirukanthan et al., 2023).

Globally, the number of species covered with genomic data is rather low (75% of orders with a species coverage below 12.87%), except for five small orders, Amiiformes (one species), Hiodontiformes (two), Lepidogalaxiiformes (one), Percopsiformes (10) and Stylephoriformes (one), where all described species have genomic data (100%; Figure 3). *Amia calva*, the only species of the order Amiiformes, is considered a living fossil given its apparent low rate of molecular evolution and morphological changes compared to the fossil record. Studying it alongside other living fossils provides a better understanding of the genome evolution, development and immunology of vertebrates (Brownstein et al., 2022). Hiodontids, along with other osteoglossomorphs, have also been used to study teleost evolution considering their basal position after genome duplication to that group (Hilton & Lavoué, 2018; Wright et al., 2022). Percopsiforms include a significant proportion of cavefish, with particular features such as the lack of vision, either by the absence of pigmentation or the eyes themselves (Dillman et al., 2011). These features are of interest for comparison with other cavefish genomes distributed in different fish orders (Zhao et al., 2022). Finally, *Stylephorus chordatus*, the only known species of Stylephoriformes, is a rare deep‐sea fish whose genome sequencing was aimed at studying the evolution of the immune system in teleost fish (Malmstrøm et al., 2016).

Semionotiformes is also a small order, yet it has a considerably high representation compared to other groups (42.86%). Species such as the spotted gar (*Lepisosteus oculatus*) are commonly used as outgroups in evolutionary, comparative and immunological studies, given their basal position to teleosts, prior to the teleost‐specific whole‐genome duplication (Braasch et al., 2016; Policarpo et al., 2025; Wu et al., 2024).

It is worth noting that since 2019, the National Genomics Data Center (NGDC), which is part of the China National Center for Bioinformation (CNCB), has been collecting multi‐omics data through its databases, including genomic data, annotations and variant information (CNCB‐NGDC Members and Partners, 2025). Although these data are integrated with the NCBI database, at least partially, it is still possible to find missing fish genomes in NGDC. In particular, the Genome Warehouse (GWH) resource of the CNCB currently contains the chromosome‐level draft genome of the species *Sillago sihama* (i.e. chromosome‐scale scaffolding that may still contain sequence gaps, local misassemblies, low‐confidence regions or unplaced scaffolds; Chain et al., 2009). This species belongs to the Sillaginidae family and currently represents the only sequenced genome available for this family. While the existence of various genome databases can improve availability, accessibility and data integrity, as they develop and potentially diverge, it will be important to generate tools to simultaneously query and integrate their information. This will allow future studies to employ the most complete datasets to answer scientific questions.

Despite great advances in fish genome sequencing, an important fraction of the bony fish phylogenetic tree remains unaccounted for (Figure 3). Five orders and 14 order‐level *incertae sedis* families lack genomic data. Most of them are percomorphs of the clades Carangaria, Ovalentaria and Eupercaria. This lack of reference genomes has to do with the poor information for some groups of the fish phylogeny. Percomorphaceae is a hyperdiverse group with a crown node age around 120 million years ago (Dornburg & Near, 2021), constituting one of the major evolutionary radiations of jawed vertebrates (Alfaro et al., 2009). The percomorph radiation is characterized by rapid diversification during the Late Cretaceous with short basal internodes (Hughes et al., 2018; Sanciangco et al., 2016). This circumstance determined the emergence of numerous lineages with extreme morphological diversity (Alfaro et al., 2018), which explains why the phylogenetical relationships within this group have been difficult to solve (Sanciangco et al., 2016). Initially, the order Perciformes, long considered a “wastebasket” based on morphological data, included most of the taxa showing no clear distinctive traits, reaching more than 10,000 species (Dornburg & Near, 2021). More recently, multilocus sequencing data identified several subclades, and now the order Perciformes is considered a monophyletic group (Betancur‐R et al., 2017). However, although the resolution of the fish tree of life has greatly improved (Hughes et al., 2018), the inclusion of more taxa and deeper genomic coverage is still needed to unravel the complex relationships among percomorphs (Hughes et al., 2018; Sanciangco et al., 2016). Recent studies have highlighted the power of whole‐genome approximations to unveil the complex evolutionary histories of some taxa (Dornburg & Near, 2021; Parey et al., 2023).

## 5 BEYOND FULLY ASSEMBLED GENOMES

**FIGURE 4:**
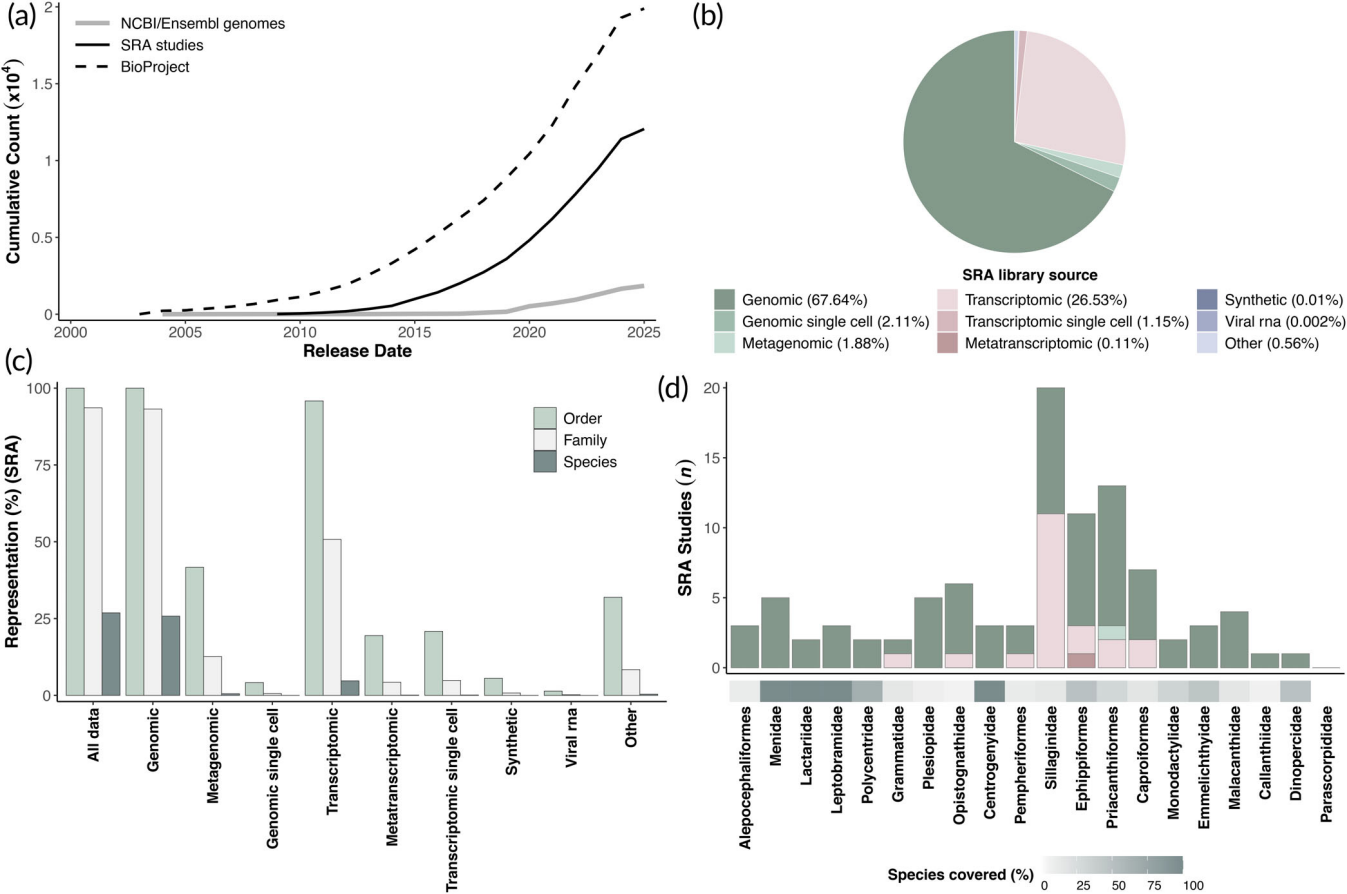
(a) Cumulative number of SRA studies, BioProjects and genomes released over time on bony fish. (b) Pie chart showing the distribution of library sources found in the SRA database. (c) Percentage of orders, families and species represented with SRA data. (d) Distribution of SRA data among those orders and families order‐level *incertae sedis* without published genomes (i.e. gaps in the bony fish phylogeny from Figure 3). The (approximate) percentage of species represented with SRA data is indicated by colour in the heatmap column. The total number of registered SRA studies per order or family, and categorized according to the library source, is indicated in the bar plot. The number of orders and families currently recognized was taken from Betancur‐R et al. (2017), and the total number of species from the Catalogue of Life.

The range of accessible genomic information for bony fish expands when we look beyond the publicly available genomes that have already been sequenced and assembled, and we delve into the raw sequencing data. Currently, the NCBI Sequence Read Archive (SRA) repository hosts the largest amount of open‐access high‐throughput sequencing data (Leinonen et al., 2010; Sayers et al., 2024). The rate at which new SRA studies are being registered far exceeds the rate at which new genomes are being published and added to the NCBI and Ensembl databases (Figure 4a). The SRA studies include a variety of library sources (Figure 4b), predominantly genomic data, mainly derived from whole‐genome sequencing (WGS) and restriction‐site associated DNA sequencing (RAD‐Seq), followed by transcriptomic data primarily generated through RNA sequencing (Files S4 and S6). Similarly, the number of BioProjects targeting bony fishes in the NCBI BioProject database far exceeds the number of SRA studies and genomes deposited (Figure 4a), with genomic and transcriptomic data being the most frequently associated material (Figure S5).

In total, SRA has data available for 100% of bony fish orders, 93.58% of families and 26.85% of recognized species, with similar values when considering only genome sequencing data (100%, 93.19% and 25.78% respectively) (Figure 4c). Remarkably, 18 out of 19 of the gaps found in the bony fish phylogeny (i.e. orders or family order‐level *incertae sedis* without published genomes in the NCBI, Ensembl or NGDC databases; Figure 4d) are filled by high‐throughput sequencing data (mainly from WGS and targeted capture sequencing), therefore it is expected that these gaps will not remain for too long. In many projects of the BioProject database the species is not specified, so the taxonomic representation is incomplete.

## 6 EVOLUTIONARY BIOLOGY FROM FISH GENOMES

While sequencing efforts have so far covered much of the phylogenetic range of bony fish, the work is far from complete. Projects like the Earth BioGenome Project (EBP) are currently underway with the ultimate goal of sequencing all eukaryotic species on earth (Lewin et al., 2018). Although this is a long way off, important advances in fish genomic resources are expected in the coming years. Filling the gaps in the fish phylogeny will provide powerful insights into the genetic basis of adaptation and genome evolution. In addition, confident genomes are essential for population genomics studies aimed at understanding local adaptation and sustainability of wild resources from a broader spectrum of organisms, decisively contributing to clarify evolutionary relationships.

Adaptation to a diverse environment is responsible for most of the phenotypic variation that we observe between and within species (Des Roches et al., 2018; Safran & Nosil, 2012). Advances in fish genomics will enable the detection of the genetic signatures responsible for adaptive processes with unprecedented precision and certainty, pinpointing the functional basis of adaptation for a myriad of traits. For instance, Xu et al. (2025) recently revealed the genomic basis of adaptation to the deep sea by sequencing 11 species covering all hadal zone lineages, and Bista et al. (2023) sequenced 24 species covering all the subgroups of the Antarctic notothenioid fish radiation, delving into the molecular adaptations that enabled the colonization of freezing seas. These studies help us understand the molecular mechanisms governing animal phenotypes, with timely practical applications. Such knowledge can help us predicting and potentially mitigating the impacts of global climate change (e.g. Bernatchez et al., 2024). Transcriptomic and epigenomic data also provide complementary information to investigate phenotypic plasticity (Johnston et al., 2024), while reference genomes enable more comprehensive, accurate and high‐resolution interpretation. Integrating multi‐omics approaches provide a greater capacity to understand population resilience.

Genomic tools are also central in conservation genomics to assess population viability and adaptive capacity. WGS data can be used to estimate inbreeding without genealogical information (e.g. McQuillan et al., 2008; VanRaden, 2008; Yang et al., 2010), which is often lacking in wild populations (Goudet et al., 2018), as well as inbreeding depression when phenotypes are available (Caballero et al., 2021). For example, studies on the nine‐spined stickleback (*Pungitius pungitius*) emphasized that severe population declines can put even large wild populations at risk by inbreeding depression (Fraimout et al., 2023). These tools can also be used to explore how population isolation and demographic history impact genetic variation and inbreeding, information that can inform restoration and management programs (Bell et al., 2025). A key parameter is the effective population size (*N*_*e*_), the main indicator for monitoring genetic diversity (Hoban et al., 2023). Multiple genomic methods can estimate both recent historical and contemporary *N*_*e*_ (e.g. Li & Durbin, 2011; Santiago et al., 2020, 2024), which have been especially applied in fish of economic relevance (e.g. Coimbra et al., 2023; Ferrette et al., 2023). For instance, studies on brown trout (*Salmo trutta*) highlighted the importance of genetic connectivity for the long‐term survival of populations with low diversity and small *N*_*e*_ (Kurland et al., 2024). Traditionally, an *N*_*e*_ of at least 500 has been considered necessary for long‐term viability (Franklin, 1980), although this value may be influenced by several factors (e.g. Caballero et al., 2017; Pérez‐Pereira et al., 2022) and the probability of reaching this threshold differs across taxa (Clarke et al., 2024). WGS also enables the estimation of the burden of deleterious mutations (e.g. Kurland et al., 2024), providing valuable insights into detrimental mutation accumulation. Altogether, data from new genomic resources will facilitate the establishment of regulations and action plans within fish conservation programs.

In terms of genome evolution, fish are a particularly interesting group due to its size (~half of all extant vertebrate species) and their unrivalled diversity (Volff, 2005). The study of tetraploidization and rediploidization events in this group has provided insights on the evolutionary dynamics of vertebrate genomes and continuous to be a flourishing area of research (Gundappa et al., 2022; Parey et al., 2022; Redmond et al., 2023). The availability of a large number of actinopterygian genomes has also enabled macroevolutionary studies to be conducted on the impact of whole‐genome duplications on the diversification of TEs (Mallik et al., 2025). Another interesting aspect of teleost fish is their remarkable diversity of sex determination systems and their fast turnover, including the rapid emergence and decay of sex chromosomes (El Taher et al., 2021; Zheng et al., 2025). The addition of high‐quality genomes for more species, especially capturing both male and female individuals, should help us finding patterns that explain the quick evolutionary rate of sex determination in teleost fish.

Long‐read sequencing technologies coupled with highly contiguous genomes are making feasible the discovery and analysis of structural variants (SVs) using new computational methods (Ahsan et al., 2023). SVs represent an essential source of genetic variation that can be used to understand adaptation and even speciation (Berdan et al., 2024; Wellenreuther & Bernatchez, 2018). The highly contiguous and increasing genomes available in fish have unveiled a large amount of SVs, including long inversions that are suggested to be involved in adaptation (Sinclair‐Waters et al., 2018; Zong et al., 2021). Even thousands of short‐ mid‐indels are being unveiled through WGS, some overlapping regulatory elements, thus with a great impact on phenotype variation (Johnston et al., 2024; Lecomte et al., 2024).

Finally, the availability of an increasing number of fish genomes will result in more precise fish phylogeny. For instance, Lü et al. (2021) could solve the polyphyletic origin of the startling body plan of flatfish (Pleuronectiformes) by analysing 11 chromosome‐level representative genomes of the order, while Parey et al. (2023) solved a long‐standing controversy about the teleost early evolution using seven high‐quality genomes. As phylogenetic relationships overarch our ability to draw conclusions about the evolution of fish species, this continuous refinement will boost all kind of evolutionary studies in teleost species. Resolving the phylogenetic relationships between organisms will also allow more precise conservation units to be established (Hohenlohe et al., 2021). For instance, Tengstedt et al. (2024) estimated an earlier divergence time than previously thought between two fish species that are the subject of long‐standing taxonomic controversy, the North Sea houting and the European lake whitefish (*Coregonus* spp.). Given the houting's inbreeding and unique genetic adaptations, they recommend a distinct conservation unit.

## 7 IMPACT OF GENOMIC RESOURCES ON FISHERIES AND AQUACULTURE

Advances in genomic tools are also revolutionizing sectors that have a direct impact on human wellbeing, such as fisheries and aquaculture. According to the Global Plan of Action for the Conservation, Sustainable Use and Development of Aquatic Genetic Resources for Food and Agriculture (FAO, 2022), several thematic areas are predicted to be largely impacted by the use of genetic technologies in the coming decade. Such areas include stock management and productivity, genetic improvement and domestication, biodiversity conservation and ecosystem impact, as well as commercial aspects (Friedman et al., 2022).

In terms of stock management and productivity, several genomic tools have the potential to improve the management, sustainability and overall performance of fisheries and farmed fish broodstock. These include (i) genetic identification and tagging of individuals from different stock populations (Andreou et al., 2012; Carruthers et al., 2023), (ii) estimation of inbreeding and relatedness coefficients among individuals (e.g. Wang, 2014; Yengo et al., 2017), (iii) age determination based on DNA methylation without sacrificing the animal (Piferrer & Anastasiadi, 2023), (iv) functional genomic analysis to identify genes involved in traits of interest (e.g. Mukiibi et al., 2025) and (v) association studies linking genes to responses to external stressors (e.g. Oikonomou et al., 2022). Estimates of genetic diversity, coupled with inbreeding coefficients for farmed populations and their wild counterparts, which could serve as future reservoirs of genetic diversity (Sonesson et al., 2023), also provide relevant information on their health status (D'ambrosio et al., 2019; Hillen et al., 2017; Villanueva et al., 2022). This makes it possible to plan breeding strategies aimed at reducing inbreeding and restore genetic diversity.

Although the field of fish genomic selection programs is relatively new compared to the livestock and crop sectors, great progress has been made in recent years (Houston et al., 2020; Sonesson et al., 2023). While genetic gains through marker‐assisted selection may be limited in the case of polygenic traits, genomic selection allows more accurate selection for traits with low heritability or those that are difficult to phenotype in breeding populations (Zenger et al., 2019). This approach relies on estimating genomic breeding values using SNPs distributed throughout the genome. Examples of species included in genomic selection programs are the Atlantic salmon (*Salmo salar*) and the rainbow trout (*Oncorhynchus mykiss*) (D'Agaro et al., 2021). Genome editing, for example through techniques such as the CRISPR/Cas9 system, is also growing rapidly (Houston et al., 2020). It has already been applied to aquaculture species such as the yellow catfish (*Silurus lanzhouensis*), the Atlantic killifish (*Fundulus heteroclitus*) and salmon (Yang et al., 2022), among others, targeting phenotypes of interest (e.g. growth, disease resistance or tolerance to stressors). However, its commercialization may be more controversial (Sonesson et al., 2023).

From the perspectives of biodiversity conservation and ecosystem impact, and in line with the previous section, estimates of abundance and *N*_*e*_ can be obtained from molecular markers to determine the demographic and genetic status of populations (Carruthers et al., 2023; Saura et al., 2021). They can also be used to evaluate the exploitation history of economically important species (Atmore et al., 2022). This information can drive programs to protect, maintain and recover high‐value species for fisheries and aquaculture. One of the recurring concerns that arises regarding farmed populations is also the possibility of fish escapes and the associated ecological and genetic impact (e.g. Bradbury et al., 2022). Integrating complete genome resources and genetic tools offers the possibility of monitoring escapees and their impact on the wild and to look for alternatives, such as generating sterile individuals (Xu et al., 2023), preventing fitness loss due to crossing between farmed and wild individuals.

However, before genetic tools can be broadly applied, several limitations must be addressed, including policy and regulatory constraints, implementation capacity and costs, and limited knowledge of species traits and biology, especially in non‐model organisms. In addition, many applications require informative molecular markers from whole or targeted genome sequencing, e.g. genotyping‐by‐sequencing (GBS) or SNP arrays, which are only available for some species (Rasal et al., 2024). Although the availability of reference genomes is not a prerequisite for obtaining informative genetic markers (e.g. RAD‐sequencing markers), high‐quality reference genomes greatly improve their identification and interpretation. At the same time, a thorough understanding of genes, how they function and how they are regulated is desirable. This can only be achieved by well‐assembled and annotated genomes, including both coding and non‐coding regions. Genomes from non‐commercial species across the fish phylogeny are also valuable, enabling comparative functional studies and potentially providing information on new candidate genes.

## 8 LOOKING FORWARD

In the last decade, fields such as ecology, evolution and conservation genomics have benefited from the impressive advances and lowering costs of sequencing technology, bioinformatic and technical assembly methods. Improvements include higher capacity of short‐read sequencing platforms, more accurate long‐read methodologies (PacBio Hi‐Fi ~ 99%) and scaffolding methods such as Hi‐C or optical mapping (Espinosa et al., 2024; Luo et al., 2021; Whibley et al., 2021). In bony fish, although the proportion of chromosomal level genome assemblies has recently increased, the majority are still at the scaffold (or contig) level (including less than 11% with contig N50 > 1 Mb), and complete genomes remain the exception.

While some applications may not require the same level of assembly quality, improvement of reference genomes will facilitate more accurate identification of genes and their regulatory elements, characterization of structural variants and complex repetitive regions. It will also allow better interpretation of recombination rates from highly dense genetic maps, improved mapping of resequencing data and, in general, a reduction in error‐proneness. Therefore, achieving complete, highly contiguous and quality assemblies is always a desirable goal. In the coming years, this will be facilitated by the constant progression of sequencing and assembling methodologies, being expected to be harnessed for evolutionary and comparative genomics of fish species. For example, the combination of PacBio HiFi, ultra‐long nanopore and Hi‐C data recently enabled the assembly of a telomere to telomere (T2T) genome for the Asian icefish (*Protosalanx chinensis*; Zhou et al., 2025), which is already accessible at the NCBI site. As noted before, reference genomes along with resequencing of individuals at population scale provide valuable insights into a broad spectrum of population genomics in teleosts. The availability of T2T genomes will also facilitate telomere‐ and centromere‐focused studies (Zhou et al., 2025). Future progress on the annotation and analysis of non‐coding genes and regulatory elements, information still incomplete among bony fish species, will also provide a broader understanding of the fish genome evolution (Iliopoulou et al., 2024).

On the other hand, the concept of pangenomes, a representation of the completeness of a species genome retrieving the most relevant intraspecific variation, has been widely investigated in prokaryotes and plants (Cummins et al., 2022; Schreiber et al., 2024). This approach addresses the limitations of using a single reference genome to understand adaptation and evolution across the whole distribution range of species (Bayer et al., 2020; Morneau, 2021; Whibley et al., 2021). Confident pangenomes rely on T2T reference genomes, now feasible with the last long‐read sequencing technologies (Chen et al., 2023; Nurk et al., 2022). Pangenomes are also becoming a target of livestock research, especially in those species cultivated in different environments worldwide or involving different strains or diverse origins. They provide a framework to understand the genetic architecture of key production traits and their interaction with the environment, such as those related to health and reproduction (Eisenstein, 2023). However, there are still major limitations not only in the feasibility of obtaining input data for each species, but also in terms of cost, current development of technologies and data storage. So far, pangenomes are available for some plant species (Bayer et al., 2020), humans (Liao et al., 2023), some domestic animals (Gong et al., 2023) and, especially, for bacteria (more than 4000 pangenomes), including some fish pathogens (Luan & Thi, 2020). Nevertheless, this picture is rapidly changing and several pangenomes are ongoing in various vertebrates, including fish.

## 9 CONCLUSION

In the last two decades, we have witnessed a genomic revolution, but fish genomics has traditionally lagged behind other vertebrates. However, in a span of just 5 years, the number of bony fish genomes sequenced and deposited in the NCBI and Ensembl databases has grown up to 1744, which implies a significant jump, representing around 5% of the described species and around 93% of the described orders. These figures, however, are not static, since these databases are constantly updated, and by the time the reader accesses these lines, the number of genomes or assemblies available will certainly have increased. As the number and quality of genome assemblies increases, it will allow broader population and comparative genomics studies, making it feasible to address outstanding questions in increasingly interconnected evolution and production research fields.

## 10 MATERIALS AND METHODS

### 10.1 Genome assemblies

The NCBI and Ensembl databases were last accessed on 23 May 2025. Both databases were scanned to record all bony fish species (ray‐finned fishes, coelacanths and lungfish) with available genomic data. The NCBI datasets command‐line tools (*datasets* and *dataformat*, version 16.40.1; O'Leary et al., 2024) were used to interrogate all genome assemblies using ‘Actinopterygii’, ‘Coelacanthimorpha’ and ‘Ceratodontiformes’ as taxon keywords. The output was first filtered to eliminate duplicate entries or entries with the status ‘suppressed’ or ‘previous’, thus keeping all genome assemblies currently available per organism (including different versions of the same assembly). Among the remaining assemblies, when possible, we retained only those categorized as the reference genome as the organism's representative. If a reference genome was not available, the assembly with the biggest scaffold or contig N50 was chosen, excluding those labelled as ‘contaminated’ with exogenous genome sequences (12 among all assemblies), ‘genome length too small’ (eight), ‘partial’ (nine) and ‘superseded by newer assembly for species’ (38). This first filtering resulted in 1846 genomes (six of them labelled as contaminated without alternative assembly and one contaminated reference genome). A second filtering was applied to separate recognized species (1738) from unclassified species within genus (80), subspecies (21), organisms classified as below‐species or with no rank (three) and hybrids (four), according to the information available in the NCBI Taxonomy Database. Only recognized species were used for summary statistics, unless the genome was the only one available for the species or genus, in which case the assembly with the biggest scaffold or contig N50 was included as the best representation of the species. Species were also replaced by an alternative representative if the latter had a reference genome, but not the former (e.g. *Gasterosteus aculeatus* replaced by the subspecies *Gasterosteus aculeatus aculeatus*). This second filtering resulted in 1759 genomes.

The Ensembl REST API (Yates et al., 2015) was used to access the latest Ensembl release 114. The Ensembl Rapid Release was also inspected via the FTP site to check for any missing genomes, although this website has been frozen since November 2024. The Ensembl Beta site (https://beta.ensembl.org/) is currently being updated with new genome assemblies, but, to our knowledge, the list of published genomes and species is not accessible, so this site has been ignored. Information related to quality statistics was read from the XML files on the European Nucleotide Archive (ENA) website, which were automatically accessed by assembly accession code. A list of all available genome assemblies was obtained by merging data from Ensembl and Ensembl Rapid Release, keeping only the Ensembl entries in case of duplicate accessions. In order to limit the data to one representative genome per organism, we applied the following filtering criteria in order of priority: (i) latest version in the case of the same assembly, (ii) chromosome level over scaffold and contig assemblies, (iii) Ensembl over Ensembl Rapid Release, (iv) bigger scaffold N50, (v) bigger contig N50 and (vi) last updated. This first filtering resulted in 266 genomes. Following the same criteria as for the NCBI, a second filtering was then applied to separate recognized species (259) from unclassified species within genus (one), subspecies (five), organisms classified as below‐species or with no rank (one) and hybrids (zero).

Finally, data from NCBI and Ensembl were merged into a single file containing all genome assemblies per organism and a second file containing all representative species (one genome per species) used for summary statistics. A last filtering was applied to the second file to remove assemblies with unusually small sequence length suspicious to be incomplete (<300 Mb; Fan et al., 2020; Nolte, 2020), and contaminated genomes without alternative assembly. When possible, contaminated genomes were replaced with the next available assembly with the largest scaffold or contig N50. Ten of the genomes that were removed were the only representatives of their genus. The final list of bony fish (representative) genomes, as well as all the assemblies available for all organisms, can be found in Files S1 and S2, respectively. These files also include taxonomic information obtained using the NCBI Datasets command‐line tools, along with assembly statistics such as genome coverage (defined as the estimated base coverage across the genome, in ‘x’ units) and contig and scaffold N50, among others. File S3 summarizes the number of assemblies available per organism.

After identifying taxonomic gaps in genomic information (see below for details on taxonomy), the Genome Warehouse (GWH) database at the China National Center for Bioinformation (CNCB) was queried to evaluate whether these gaps could be partially filled. Because GWH contains both assemblies integrated from NCBI and assemblies submitted directly by users, only the latter were reviewed. However, current search and data retrieval limitations prevented the systematic and automated extraction of assembly quality statistics. Therefore, GWH was not included in the overall statistical analyses, and only genomes from species filling previously identified taxonomic gaps are reported.

### 10.2 High‐throughput sequencing data and research projects in bony fish

Information on all available raw sequencing data and BioProjects focused on bony fish was retrieved from the SRA and BioProject databases. The SRA database was last accessed between 10 and 16 April 2025, and the BioProject database on 7 May 2025. For both databases, the commands *esearch* and *efetch* from the NCBI Entrez Direct (EDirect; Kans, 2024) were used to download complete lists of SRA runs and BioProjects. ‘Actinopterygii’, ‘Ceratodontiformes’ and ‘Coelacanthimorpha’ were used as the query organisms. Due to server errors, smaller searches were performed using each of the taxonomic orders within the superclass as a separate query in the case of SRA data for actinopterygians. The complete list of SRA entries and BioProjects can be found in Files S4 and S5, respectively, along with taxonomic information. The global number of SRA studies, experiments and runs per library source and organism is summarized in File S6. Similarly, the global number of BioProjects per target material and organism is summarized in File S7.

SRA entries were recorded for a total of 10,137 organisms, including species (8697; six of which were species specifications for ploidy level or geographic location), subspecies (94), unclassified or unidentified species (1144), categorized as below species or with no rank (10), hybrids (103) and other higher taxonomic categories in which the organism was not specified (88, including orders, families, subfamilies and genus). One amphitriploid of *Carassius gibelio*, *Carassius auratus* and *Carassius cuvieri* was also recorded. In order to follow a similar criterion to that used with the NCBI and Ensembl genomes, statistics were obtained from the representative species. The previous categories were only included in the list of representative species if they were the only representant of the species, genus, family or order within the data, ignoring specifications for ploidy level, geographic location or variant name.

A total of 4890 organisms, four environmental samples and 5287 BioProjects without organism identification were extracted from the BioProject database. The organisms included species (4546, three of which corresponded to species with specification of ploidy level or geographical location), subspecies (68), unclassified or unidentified species (73), categorized as below species or with no rank (seven), hybrids (108) and other higher taxonomic categories (86). The *Carassius* amphitriploid and one tetraploid intergeneric hybrid carp were also recorded. A total of 285 organisms were covered under umbrella projects, in addition to 17 umbrella projects without organism identification. Representation statistics were obtained from the representative species following the same criteria as for SRA data.

### 10.3 Bony fish taxonomy

Both the nomenclature and the taxonomic classification were taken from the NCBI (Sayers et al., 2024) for practical purposes. The fish tree by Betancur‐R et al. (2017) was taken as reference of the phylogenetic relationships among taxa. It should be noted that the NCBI adopted previous versions of Betancur‐R and colleagues' classification (Betancur‐R et al., 2017), although some discrepancies can be found between them. To obtain an estimate of the proportion of species with genomic data compared to the total number of species currently recognized, we accessed the Catalogue of Life (FishBase as data source; https://www.catalogueoflife.org/). Due to discrepancies in the classification of orders between the Catalogue and the NCBI (orders not being recognized in the former and different distribution of families among orders), the total number of species within an order was calculated as the sum of species from each of the families that make up the order according to the NCBI. Any discrepancy at the level of genus that makes up a family was ignored. Therefore, these estimates should be taken as approximations. When the number of genomes exceeded the number of recognized species, the genome representation was adjusted to 100%.

## AUTHOR CONTRIBUTIONS

Conceptualization: N.P.P. and D.R. Database review: N.P.P. Writing – original draft: N.P.P. Writing – review and editing: all authors. Funding: D.R.

## FUNDING INFORMATION

European Union ERC Starting Grant programme 2022 No 101076432 (FishTRIM). Oportunius programme of the Axencia Galega the Innovación (GAIN, Xunta de Galicia). BBSRC Institute Strategic Grants to the Roslin Institute (BBS/E/RL/230001A and BBS/E/RL/230002A). Galicia Marine Science programme, which is part of Complementary Science Plans for Marine Science of Ministerio de Ciencia, Innovación y Universidades included in the Recovery, Transformation and Resilience Plan (PRTR‐C17.I1) co‐funded by Xunta de Galicia Next Generation EU and the European Maritime Fisheries and Aquaculture Funds. Regional Government Xunta de Galicia (Spain) program (Grant number ED431C 2022/33). Funding for open access charge: Universidade de Santiago de Compostela/CISUG.

## CONFLICT OF INTEREST STATEMENT

The authors declare no conflict of interest.

## Supporting information


**FIGURE S1.** Boxplots showing genome size variation of bony fish. Total sequence length in gigabases (Gb). Orders and order‐level *incertae sedis* sorted according to the phylogenetic tree by Betancur‐R et al. (2017), following Figure 3 of the main text. Taxa without genomic data were removed. Boxes represent the interquartile range, and the median is indicated by a solid line. Note the *x*‐axis break separating Ceratodontiformes from other bony fish (*ggbreak* by Xu et al., 2021).
**FIGURE S2.** Boxplots showing variation of haploid chromosome number in bony fish. Orders and order‐level *incertae sedis* sorted according to the phylogenetic tree by Betancur‐R et al. (2017), following Figure 3 of the main text. Taxa without genomic data were removed. Boxes represent the interquartile range, and the median is indicated by a solid line.
**FIGURE S3.** Boxplots showing variation in the number of protein‐coding genes, non‐coding genes and pseudogenes. Orders and order‐level *incertae sedis* sorted according to the phylogenetic tree by Betancur‐R et al. (2017), following Figure 3 of the main text and excluding taxa without genomic data. Boxes represent the interquartile range, and the median is indicated by the solid line.
**FIGURE S4.** Assembly quality, measured as contig N50 (Mb), for each of the genomes deposited at National Center for Biotechnology Information and Ensembl (Rapid Release) per organism. Only organisms with five or more genomes are shown. The boxes represent 50% of the estimates, i.e. the interquartile range, and the median is indicated by the horizontal solid line. Each circle represents a genome (reference genome in red). The total number of genomes per organism is also indicated in parentheses.
**FIGURE S5.** (a) Cumulative number of Sequence Read Archive studies, bioprojects and genomes released over time on bony fish (Figure 4a in the main text). (b) Pie chart showing the distribution of library materials found in the BioProject database. (c) Percentage of orders, families and species represented with associated BioProjects. (d) Distribution of BioProjects among those orders and families order‐level *incertae sedis* without published genomes (i.e. gaps in the bony fish phylogeny from Figure 3). The (approximate) percentage of species represented with BioProjects is indicated by colour in the heatmap column. The total number of registered BioProjects per order or family, and categorized according to the library material, is indicated in the bar plot. The number of orders and families currently recognized were taken from Betancur‐R et al. (2017), and the total number of species from the Catalogue of Life.

**SUPPORTING INFORMATION FILE S1.** Representative bony fish genomes found in the National Center for Biotechnology Information and Ensembl databases.

**SUPPORTING INFORMATION FILE S2.** Genome assemblies available per organism in the National Center for Biotechnology Information and Ensembl databases.

**SUPPORTING INFORMATION FILE S3.** Number of assemblies available for the organisms found in the National Center for Biotechnology Information and Ensembl databases.

**SUPPORTING INFORMATION FILE S4.** List of Sequence Read Archive entries for bony fish.

**SUPPORTING INFORMATION FILE S5.** List of BioProjects for bony fish.

**SUPPORTING INFORMATION FILE S6.** Global number of Sequence Read Archive studies, experiments and runs per library source and organism.

**SUPPORTING INFORMATION FILE S7.** Global number of BioProjects per target material and organism.

## Data Availability

Scripts for this study are available at Github address https://github.com/noeliaperezp/FishGenomes.
