# Supplementary material for: Bony fish genomes: Status and gaps
Source: J Fish Biol. 2026 Mar 18;109(2):631–49. doi: 10.1111/jfb.70402 (PMC13521744; doi:10.1111/jfb.70402)
Supplement: Supplementary file 1 [file JFB-109-631-s007.docx]

**SUPPLEMENTAL FIGURES**

**Figure S1.** Boxplots showing genome size variation of bony fish. Total sequence length in gigabases (Gb). Orders and order-level *incertae sedis* sorted according to the phylogenetic tree by Betancur-R et al. (2017), following Fig. 3 of the main text. Taxa without genomic data were removed. Boxes represent the interquartile range, and the median is indicated by a solid line. Note the x-axis break separating Ceratodontiformes from other bony fish (*ggbreak* by Xu et al., 2021).

**Figure S2.** Boxplots showing variation of haploid chromosome number in bony fish. Orders and order-level *incertae sedis* sorted according to the phylogenetic tree by Betancur-R et al. (2017), following Fig. 3 of the main text. Taxa without genomic data were removed. Boxes represent the interquartile range, and the median is indicated by a solid line.

**Figure S3.** Boxplots showing variation in the number of protein-coding genes, non-coding genes and pseudogenes. Orders and order-level *incertae sedis* sorted according to the phylogenetic tree by Betancur-R et al. (2017), following Fig. 3 of the main text and excluding taxa without genomic data. Boxes represent the interquartile range, and the median is indicated by the solid line.

**Figure S4.** Assembly quality, measured as contig N50 (Mb), for each of the genomes deposited at NCBI and Ensembl (Rapid Release) per organism. Only organisms with five or more genomes are shown. The boxes represent the 50% of the estimates, i.e., the interquartile range, and the median is indicated by the horizontal solid line. Each circle represents a genome (reference genome in red). The total number of genomes per organism is also indicated in parentheses.

**Figure S5.** A) Cumulative number of SRA studies, bioprojects and genomes released over time on bony fish (Fig.4A in the main text). B) Pie chart showing the distribution of library materials found in the BioProject database. C) Percentage of orders, families and species represented with associated BioProjects. D) Distribution of BioProjects among those orders and families order-level *incertae sedis* without published genomes (i.e., gaps in the bony fish phylogeny from Fig. 3). The (approximate) percentage of species represented with BioProjects is indicated by colour in the heatmap column. The total number of registered BioProjects per order or family, and categorized according to the library material, is indicated in the bar plot. The number of orders and families currently recognized were taken from Betancur-R et al. (2017), and the total number of species from the Catalogue of Life.

**References**

Xu, S., Chen, M., Feng, T., Zhan, L., Zhou, L., & Yu, G. (2021). Use ggbreak to effectively utilize plotting space to deal with large datasets and outliers. Frontiers in Genetics, 12, 774846. https://doi.org/10.3389/fgene.2021.774846
